# Collagen XII mediated cellular and extracellular mechanisms in development, regeneration, and disease

**DOI:** 10.3389/fcell.2023.1129000

**Published:** 2023-03-02

**Authors:** Yayoi Izu, David E. Birk

**Affiliations:** ^1^ Department of Laboratory Animal Science, Faculty of Veterinary Medicine, Okayama University of Science, Imabari, Japan; ^2^ Department of Orthopaedic Surgery, Perelman School of Medicine, University of Pennsylvania, Philadelphia, PA, United States

**Keywords:** collagen XII, myopathic EDS, cell-cell communication, development, regeneration

## Abstract

Collagen XII, a fibril-associated collagen with interrupted triple helices (FACIT), influences fibrillogenesis in numerous tissues. In addition to this extracellular function, collagen XII also directly regulates cellular function. Collagen XII is widely expressed in connective tissues, particularly tendons, ligaments, and the periodontium and periosteum, where it is enriched in the pericellular regions. Mutations in the collagen XII gene cause myopathic Ehlers-Danlos syndrome (mEDS), an early-onset disease characterized by overlapping connective tissue abnormalities and muscle weakness. Patients with mEDS exhibit delayed motor development, muscle weakness, joint laxity, hypermobility, joint contractures, and abnormal wound healing. A mEDS mouse model was generated by deletion of the *Col12a1* gene, resulting in skeletal and muscle abnormalities with disorganized tissue structures and altered mechanical properties. Extracellularly, collagen XII interacts with collagen I fibrils and regulates collagen fibril spacing and assembly during fibrillogenesis. Evidence for the binding of collagen XII to other EDS-related molecules (e.g., decorin and tenascin X) suggests that disruption of ECM molecular interactions is one of the causes of connective tissue pathology in mEDS. Collagen XII also has been shown to influence cell behavior, such as cell shape and cell-cell communication, by providing physical connection between adjacent cells during tissue development and regeneration. The focus of this review is on the functions of collagen XII in development, regeneration, and disease.

## Introduction

**FIGURE 1:**
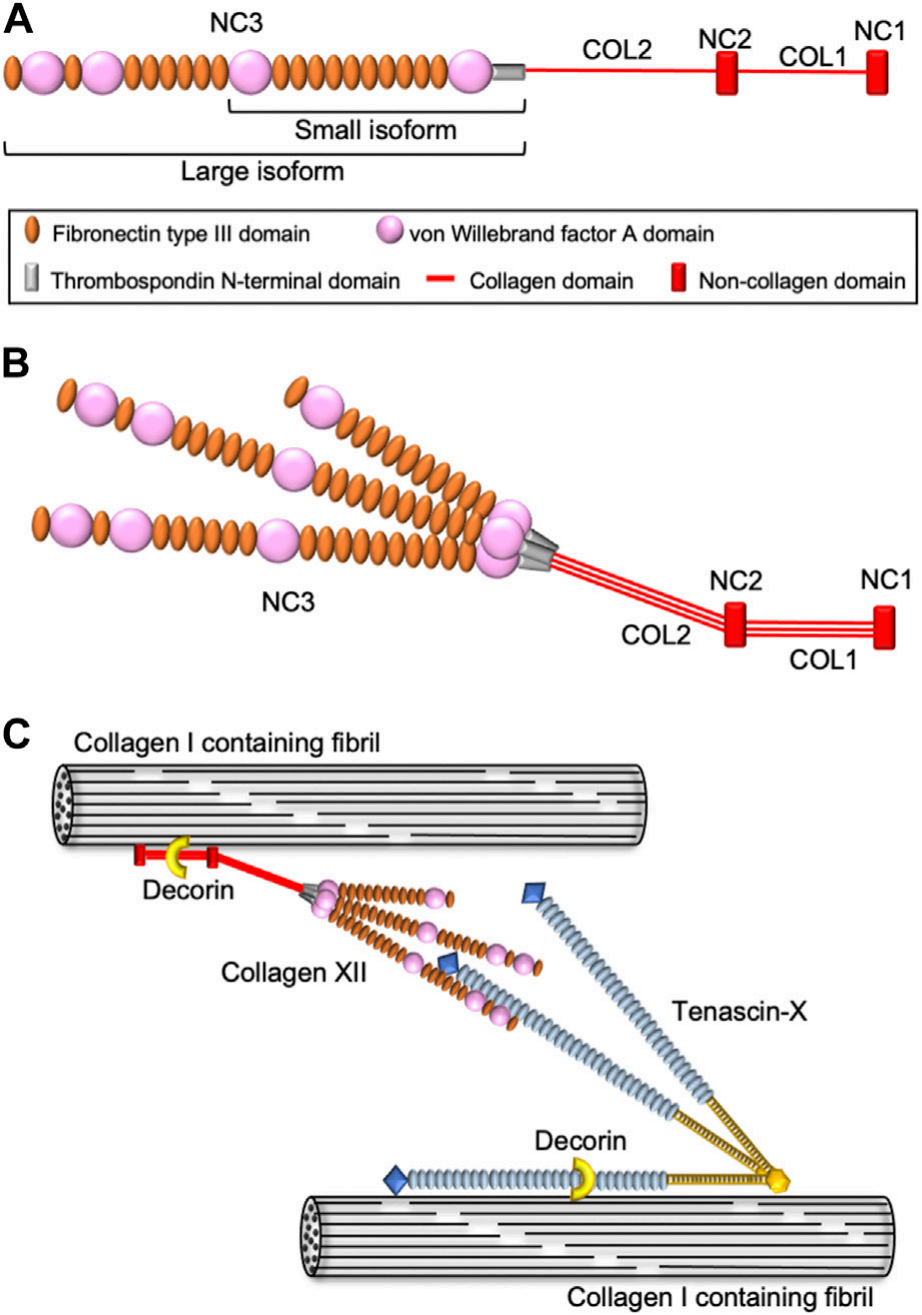
Schematic diagram of collagen XII structure and molecular interactions. **(A)** Domain structure of collagen XII. Collagen XII consists of two short triple helical domains (COL1 and COL2) separated by non-collagenous domains (NC1 and NC2) and large amino-terminal non-collagenous domains (NC3). NC3 is composed of a thrombospondin N-terminal domain (TSPN), four von Willebrand factor A domains, and 18 fibronectin type III repeats. **(B)** The heterotrimeric structure model of collagen XII consisting of two large isoforms (XIIA) and one short isoform (XIIB). **(C)** Hypothetical model for collagen XII extracellular interaction with other ECM molecules. Collagen XII interacts with the collagen I-containing fibril surface *via* its collagenous domain. Decorin might involve in this interaction in a glycosaminoglycan dependent manner. Large NC3 domain of collagen XII interact with tenascin-X, which binding to collagen I-containing fibrils is mediated by decorin.

Collagen XII belongs to the fibril-associated collagen with interrupted triple helices (FACIT) family and is an α1 homotrimer consisting of two short collagenous domains and three non-collagenous domains, including a large N-terminus domain (Koch et al., 1992; Dublet et al., 1989) (Figure 1A). Collagen XII has variants in the NC1 and NC3 domains generating four different isoforms. Major splice variants in the NC3 domain result in large XIIA and small XIIB isoforms of collagen XII (Trueb and Trueb, 1992; Chiquet et al., 2014). A large XIIA NC3 domain consists of 18 fibronectin type III (FN3) repeats with four von Willebrand factor A (vWA) modules, whereas the small XIIB isoform lacks half of the NC3 domain. Interestingly, collagen XII molecules assemble as homotrimers as well as XIIA and XIIB heterotrimers (Koch et al., 1995; Chiquet et al., 2014) (Figure 1B). Both large and small variants are present in humans and mice, and their expression differs depending on developmental stage and tissue localization. After birth, the small variants become predominant, and large variants are restricted to dense connective tissues such as tendons, ligaments, periodontium, and periosteum (Böhme et al., 1995; Karimbux and Nishimura, 1995). Alternative splice variants at the 3′-end generate variants encoded by exon 1 or 2, respectively. The long NC1 variant shares a sequence with collagen XIV, another FACIT structurally similar to collagen XII. The collagen XIV domain contains a glycosaminoglycan binding sequence, and the long NC1 variant is believed to function in the interaction with glycosaminoglycans (Kania et al., 1999). On the other hand, the short NC1 variant shares a common sequence with collagen IX, which contains information for alpha chain selection and triple helix assembly (Bon et al., 2003). Because these NC1 variants differ in both structure and tissue expression, each variant is considered to have a tissue- or event-specific function.

Collagen XII is believed to be responsive to mechanical stress. This is supported by evidence that collagen XII was induced by tensile or cyclic strain in fibroblastic cells, and it was shed when the stress was removed (Trächslin et al., 1999; Arai et al., 2008). Furthermore, the chicken *Col12a1* promoter has an enhancer region that responds to static tensile strain (Chiquet et al., 1998). This evidence suggests that collagen XII forms ECM complexes that absorb or transduce mechanical signals in response to mechanical stress. Future studies should investigate the detailed molecular mechanisms involved in this process. Other functions of collagen XII have been elucidated, especially with the discovery of *COL12A1*-related disease.

## *COL12A1* mutations in human diseases

The first patients harboring *COL12A1* mutations were reported in 2014 (Hicks et al., 2014; Zou et al., 2014). Since then, 16 families have been reported, with both autosomal dominant and autosomal recessive inheritance (Malfait et al., 2017; Punetha et al., 2017; Witting et al., 2018; Mohassel et al., 2019; Delbaere et al., 2020; Araújo and Antunes, 2021; Coppens et al., 2022). The individuals present with Ehlers-Danlos syndrome (EDS)-like symptoms, such as distal joint hypermobility in combination with proximal joint contractures, and abnormal scarring, as well as myopathic features including muscle hypotonia and weakness with delayed motor development. Based on the symptoms, the *COL12A1*-related disease was classified as myopathic type Ehlers-Danlos syndrome (mEDS) in 2017 (Malfait et al., 2017). *COL12A1* variants homozygous for loss-of-function mutations have been reported to cluster in the hinge region located in the transition between the NC3 domain and fibril-associated region, that includes the TSPN and COL2 domain (Delbaere et al., 2020). The collagenous domains are involved in fibrillogenesis with binding to collagen I and COMP (Keene et al., 1991; Koch et al., 1995; Agarwal et al., 2012), whereas the TSPN domain appears to regulate collagen XII secretion since mutations in this domain are associated with accumulated intracellular collagen XII (Delbaere et al., 2020). In addition, collagen XII also interacts with other extracellular molecules, such as decorin, fibromodulin (Font et al., 1996), and Tenascin X (Veit et al., 2006) (Figure 1C), that also are involved in fibrillogenesis and EDS pathologies. Indeed, some mEDS patients have altered expression of these ECM molecules. Understanding the relationship between different variants and the pathology will allow for the elucidation of the function(s) of collagen XII and provide a foundation for development of future treatments.

The generation of genetically modified *Col12a1* animals has allowed further definition of the functions of collagen XII (Izu et al., 2011; Agarwal et al., 2012; Hemmavanh et al., 2013; Marro et al., 2016; Wehner et al., 2017; Schönborn et al., 2020; Sun et al., 2020; Fukusato et al., 2021; Izu et al., 2021; Fung et al., 2022). A mouse with a 2–5 exon deletion in the *Col12a1* gene yielded a conventional null for collagen XII (Izu et al., 2011). The *Col12a1* null mice were small and demonstrated kyphosis, abnormal skeletal development with decreased muscle volume, abnormal gait, and decreased grip strength, comparable with the clinical presentation seen in mEDS patients (Izu et al., 2011; Zou et al., 2014). In addition, *Col12a1* overexpression under the control of *the Col1a2* promoter (Schönborn et al., 2020) and a tendon-specific conditional *Col12a1* deletion in mice have been developed (Fung et al., 2022). Analysis of tendons, bones, skin, and corneas from genetically modified *Col12a1* mice demonstrated that collagen XII is involved in fibrillogenesis, regulating collagen I fibril spacing and assembly, clearly indicating an extracellular function of collagen XII in fibrillogenesis, tissue structure and function.

The evidence from analysis of mEDS pathologies and animal models clearly demonstrate that the extracellular interaction between collagen XII and other ECM molecules involved in fibrillogenesis, tissue structure, and mechanotransduction are necessary for functional tissue development. In addition to such extracellular functions, collagen XII has been demonstrated to regulate cell shape and cell-cell communication by providing physical connections between adjacent cells with collagen bridges (Izu et al., 2011; Izu et al., 2016; Wehner et al., 2017; Izu et al., 2021). Next, we focus on the direct regulation of collagen XII on cell behavior during tissue development and regeneration.

## Collagen XII regulates cell-cell communication *via* collagen bridge formation

Collagen XII expression is ubiquitous in collagen I-containing mesenchymal tissues during embryonic development (Böhme et al., 1995; Bader et al., 2009). After birth, its distribution becomes more restricted, i.e., tendon sheath, periosteum and periodontium (Karimbux and Nishimura, 1995; Reichenberger et al., 2000). At the cellular level, collagen XII is enriched in the pericellular region, where cell-cell communication occurs (Izu et al., 2016; Izu et al., 2021).

**FIGURE 2:**
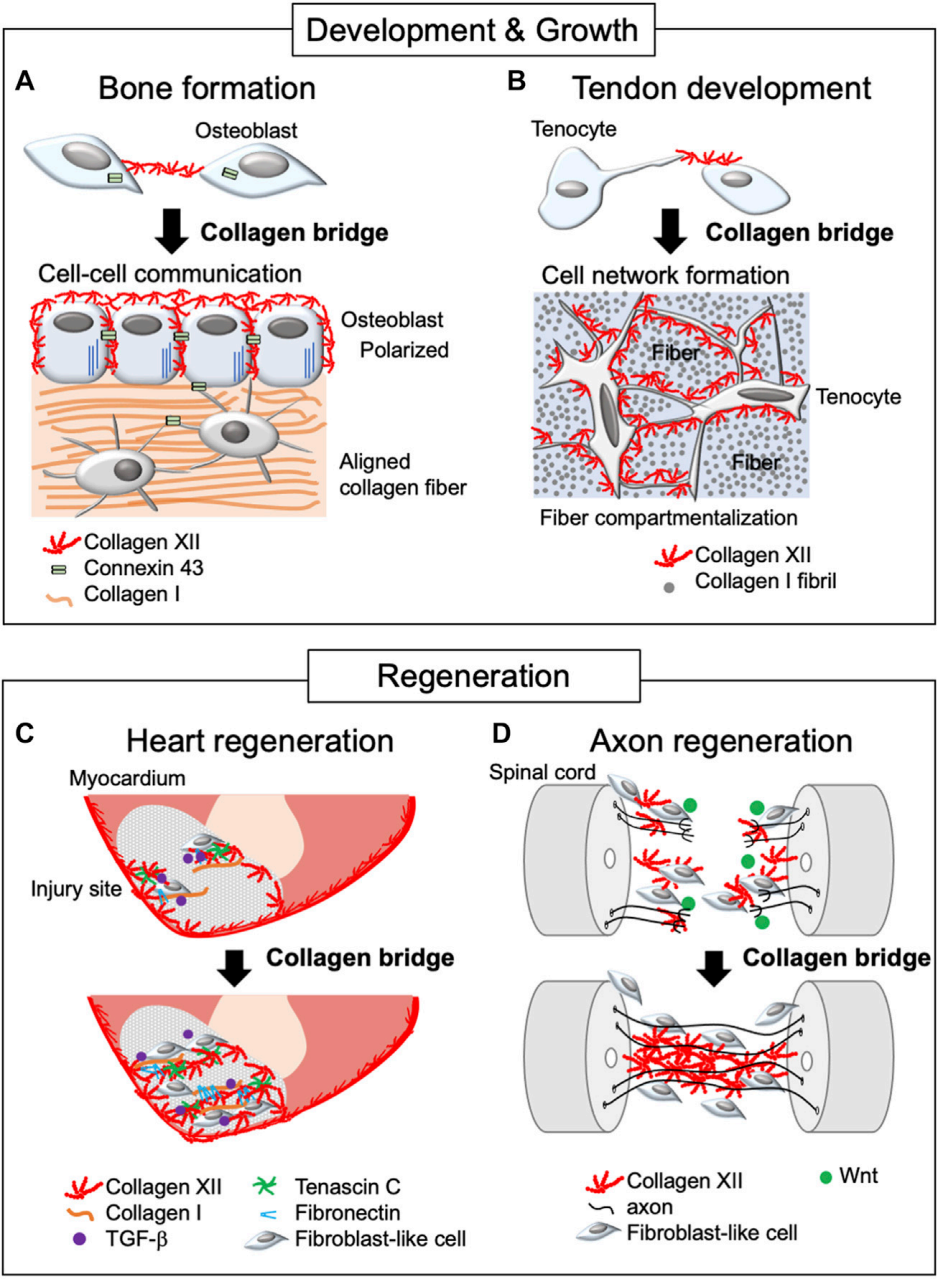
Schematic diagram of collagen XII bridge formation during development, growth, and regeneration. The diagram illustrates bone formation **(A)**, tendon development **(B)**, and heart **(C)** and axon regeneration **(D)**. Collagen XII bridges are formed between adjacent cells during bone formation and tendon development, making intercellular network formation necessary for developing functional tissue. In the regeneration process, collagen XII accumulation is found at the leading edge of the injured sites and bridges between truncated tissues, allowing cell migration. This is under the control of TGF-β and Wnt/β-catenin signaling in zebrafish during heart and axon regeneration, respectively.

During the process of bone formation, mature osteoblasts are responsible for the deposition of the bone matrix, thus, in the periosteum and endosteum, coordination of osteoblast shape, orientation, physical interaction with neighboring cells, and formation of a communicating networks *via* cadherins and connexins are critical. These properties are critical for healthy and strong bone formation (Ilvesaro et al., 1999). Collagen XII is expressed in both the periosteum and endosteum and is localized around mature osteoblasts (Böhme et al., 1995; Izu et al., 2011). In *Col12a1* null mice, mature osteoblasts had altered shape with poor polarization, and impaired cell-cell communication *via* gap junctions. *Col12a1* null mice exhibited decreased bone mass with a disorganized bone structure. Studies using *in vitro* primary osteoblast cultures demonstrated that collagen XII appeared on the cell surface and thereafter extended toward neighboring cells to form collagen bridges (Izu et al., 2016). This suggests that the physical connection of collagen XII bridges between neighboring osteoblasts is essential for acquiring and maintaining mature osteoblast bone-forming ability (Figure 2A). It is noteworthy that collagen bridge formation also requires collagen VI because a deficiency of either collagen XII or VI disrupts the formation of collagen bridges. Patients with *COL6A1* mutations suffer Bethlem myopathy and Ullrich congenital muscular dystrophy, which are clinically similar to mEDS (Hicks et al., 2014; Zou et al., 2014), suggesting functional interactions. Although collagens VI and XII are co-localized at the cell culture level, their direct interactions have not been defined. Moreover, the changes in collagen VI or XII expression in the patients with *COL6A1* or *COL12A1* mutations remain controversial (Hicks et al., 2014; Punetha et al., 2017; Delbaere et al., 2020), and further studies are required.

Collagen XII bridge formation also has been observed in primary tenocyte cultures (Izu et al., 2021). Tendon is a highly organized tissue and development of organized tendon ECM involves several compartments, including collagen fibril and fiber forming compartments, which, together with adjacent tenocytes, are organized into a fascicle (Birk and Trelstad, 1984; Birk and Zycband, 1994; Graham et al., 2000; Canty and Kadler, 2005; Zhang et al., 2005). Developing a hierarchically organized tendon structure requires columnar alignment of tenocytes along the longitudinal axis and tenocyte network formation between laterally adjacent tenocytes *via* cell process extension perpendicular to the longitudinal axis. The primary unit comprises fibrils that organize into fibers, surrounded by tenocytes and fascicular ECM, where collagen XII is expressed (Zhang et al., 2003; Izu et al., 2021) (Figure 2B). As with osteoblasts, the altered tenocyte shape, and impaired cell-cell connection/communication *via* connexin 43 found in *Col12a1* null mice may be due to the lack of collagen XII bridge formation between cells. This alteration is associated with less compartmentalized as well as disorganized tendon structure, altered tendon mechanical properties (Izu et al., 2021; Fung et al., 2022), and increased risk of articular cruciate ligament injury (Fukusato et al., 2021). These data indicate the essential role of collagen XII bridge formation during tenocyte network formation and coordination of the tendon hierarchical structure and mechanical properties. Thus, collagen XII plays a role in establishing intercellular communication by creating collagen bridges between neighboring cells during tissue development and growth.

## The role of collagen XII during tissue repair and regeneration

In addition to its roles in development and growth, collagen XII also is involved in tissue repair and regeneration. Key roles in repair and regeneration are supported by studies in mice (Schönborn et al., 2020) and in other species with high regenerative capacity, such as salamander (Zukor et al., 2011) and zebrafish (Marro et al., 2016; Wehner et al., 2017; Bise et al., 2020; Bretaud et al., 2021).

Cryoinjury in the zebrafish heart has been used as a human regeneration model for post myocardial infarction. Cryoinjury destroys cardiac cells, but preserves the collagenous layer of the epicardium, where collagen XII is developmentally and physiologically localized. During the repair process, dynamic accumulation of collagen XII was observed at the injury sites (Marro et al., 2016; Bise et al., 2020; Bretaud et al., 2021) (Figure 2C). Interestingly, collagen XII was found at the regenerative leading edge of cardiomyocytes, bridging the provisional fibrotic tissue. At the regenerative leading edge, collagen XII interacts with tenascin C, collagen I, and fibronectin, where TGF-β signaling is activated. On the other hand, TGF-β inhibitor blocked cardiomyocyte recruitment together with the extracellular matrix, including collagen XII, suggesting that cardiomyocytes induce collagen XII expression *via* the TGF-β signaling pathway. Also, collagen XII has been demonstrated to sequester latent, but not active TGF-β during corneal remodeling (Sun et al., 2021) and skin repair (Schönborn et al., 2020). Extracellular interactions between collagen XII and TGF-β regulating cells may differ depending on the tissue as well as developmental and pathological phases, and future studies will be needed.

In addition, collagen XII accumulation promoted axon growth across the lesion site during functional recovery in a zebrafish model of spinal cord injury (Wehner et al., 2017). The lesion-specific *Col12a1* transcription and deposition of collagen XII are regulated by fibroblast-like cells through Wnt/β-catenin signaling. Wnt/β-catenin signaling also regulates the expression of *Col6a2*, which encodes the collagen VI α-chain, together with *Col12a1a/b* spinal cord regeneration. This is consistent with the colocalization of collagens VI and XII in osteoblast network formation (Izu et al., 2016). Most strikingly, *Col12a1a* overexpression was sufficient for axon regeneration without Wnt/β-catenin signaling, suggesting that a physical bridge *via* collagen XII between truncated sites provides a critical microenvironment for axon growth and cells responsible for regeneration (Figure 2D).

Similar to regeneration, the accumulation and bridge formation of collagen XII are found in wounded skin, but it seems to promote fibrosis and scarring (Moinzadeh et al., 2013; Bergmeier et al., 2018). In support of this, the free-living African spiny mouse, that suppresses collagen XII induction at skin wound sites, can regenerate skin after injury without scarring due to lack of macrophage infiltration (Seifert et al., 2012; Brant et al., 2016). Surprisingly, however, excisional wounds were not repaired in either mouse models lacking or overexpressing collagen XII (Schönborn et al., 2020). In the *Col12a1* overexpressing mice, only inflammatory M1 macrophages, but not M0 or M2 macrophages, were upregulated in adhesion to collagen XII, suggesting that excess collagen XII may activate M1 macrophages *via* specific binding sites. Thus, collagen XII bridge formation is involved in tissue development, regeneration, and repair; however, the outcome of tissue remodeling may differ depending on the collagen XII-binding cells.

## Conclusion

Identification of *COL12A1* mutations in human patients and generation of genetically modified animal models have contributed to the understanding of the functional roles of collagen XII. These studies demonstrated that collagen XII is involved in both regulation of extracellular organization and cellular behavior necessary for tissue structure and function. Accumulating evidence demonstrates an essential function for collagen XII in bridge formation during tissue development, regeneration, and repair. This would be considered a new regulatory system for cell behaviors, such as cell polarization, migration, and cell-cell communication. Collagen XII bridge formation requires collagen VI and interactions with other ECM molecules such as tenascin C, tenascin X, fibronectin, and decorin. However, because collagen XII appears at the leading edge of the transected tissues or processes of distant cells, specific receptors for collagen XII may exist and function at the cell surface rather functioning *via* other ECM molecules. Induction of collagen XII expression seems to be regulated by Wnt/β-catenin and TGF-β/SMAD signaling during tissue regeneration in zebrafish, but because the receptors for collagen XII have not been found, the upstream and downstream signaling of collagen XII are still controversial. Finding a binding counterpart would be the next challenge in understanding the mechanisms of collagen XII. This will advance our understanding of the pathogenesis of mEDS and contribute to the establishment of a treatment for the disease.
